# Novel imaging and clinical phenotypes of CONDSIAS disorder caused by a homozygous frameshift variant of *ADPRHL2*: a case report

**DOI:** 10.1186/s12883-020-01873-3

**Published:** 2020-08-03

**Authors:** Hajar Aryan, Ehsan Razmara, Dariush Farhud, Marjan Zarif-Yeganeh, Shaghayegh Zokaei, Seyed Abbas Hassani, Mahmoud Reza Ashrafi, Masoud Garshasbi, Ali Reza Tavasoli

**Affiliations:** 1 National Institute of Genetic Engineering and Biotechnology, Tehran, Iran; 2 Dr. Farhud’s Genetics Clinic, Tehran, Iran; 3 Department of Medical Genetics, Faculty of Medical Sciences, Tarbiat Modares University, Tehran, Iran; 4 School of Public Health, Tehran University of Medical Sciences, Tehran, Iran; 5 Department of Basic Sciences, Iranian Academy of Medical Sciences, Tehran, Iran; 6 Cellular and Molecular Endocrine Research Center, Research Institute of Endocrine Sciences, Shahid Beheshti University of Medical Sciences, Tehran, Iran; 7 School of Advanced Medical Science, Islamic Azad University, Tehran, Iran; 8 Pediatric Intensive Care Medicine Department, Children’s Medical Center, Tehran University of Medical Sciences, Tehran, Iran; 9 Myelin Disorders Clinic, Pediatric Neurology Division, Children’s Medical Center, Pediatrics Center of Excellence, Tehran University of Medical Sciences, Tehran, Iran

**Keywords:** ADP-ribosylhydrolase, CONDSIAS, Neurodegeneration disease, Novel phenotypes

## Abstract

**Background:**

Stress-induced childhood-onset neurodegeneration with variable ataxia and seizures (CONDSIAS) is an autosomal recessive disorder caused by defects in the *ADP-Ribosylhydrolase Like 2* (*ADPRHL2*; OMIM: 618170) gene. This gene encodes the ADP-ribosylhydrolase enzyme (ARH3) that eliminates the addition of poly-ADP ribose (PAR) in the cellular stress onto proteins in the ADP-ribosylation process in which adding one or more ADP-ribose moieties onto the target proteins in the post-translational modification have occurred. In this study, we report a new case of CONDSIAS in the Iranian population. A literature review of CONDSIAS is also included.

**Case presentation:**

A four-year-old female patient, born to a consanguineous Iranian family, was referred with various clinical symptoms including impaired speech, variable ataxia, infrequent seizures, and gradual onset of truncal hypotonia. Over time, she developed complete motor and speech regression, bilateral sensorineural hearing loss, infrequent seizures, abdominal distension and gastrointestinal (GI) intolerance, and loss of consciousness. To better molecularly diagnose, trio-whole-exome sequencing (WES) was performed on the proband and her parents. Sanger sequencing was also applied to investigate co-segregation analysis. Using in silico predictive tools, the possible impacts of the variant on the structure and function of ADPRHL2 protein were predicted. All basic metabolic tests were normal, while serial coronal magnetic resonance imaging (MRI) showed progressive cerebral and cerebellar atrophy in addition to cerebral white matter signal changes as a novel neuroimaging finding. GI intolerance was another novelty of clinical scenarios in the patient. An auditory brainstem response test showed a severe bilateral sensorineural hearing loss. An electroencephalogram also confirmed focal seizures. From the molecular perspective, a novel homozygous frameshift variant in the *ADPRHL2* gene (NM_017825.2; c.636_639del, p.(Leu212fs)) was identified by WES.

**Conclusions:**

CONDSIAS is an ultra-rare neurodegenerative disorder. In the present study, we introduced extra-neurological and neuroimaging findings of this disorder in a female child caused by a novel frameshift variation in the *ADPRHL2* gene.

## Background

ADP-ribosylation is an important post-translational modification in many physiological pathways, e.g. DNA repair, transcription, translation, and apoptosis [1–4]. In this post-translational modification, the ADP-ribose units are transported from nicotinamide adenine dinucleotide (NAD^+^) onto the target protein by ADP-ribosyltransferases and added to a poly-ADP-ribose (PAR) sequence; this process was firstly suggested by Chambon et al. in 1963 [5, 6]. It was demonstrated that the poly-ADP-ribose levels may be increased 10- to 500-folds in response to genotoxic stress, oxidative stress, or mitogenic stimuli [7]. This can prevent cell death in the cellular stress settings, although its over-accumulation, in turn, leads to cell death. To address this, the *ADPRHL2* (ADP-Ribosylhydrolase like 2; NM_017825.2; MIM: 610624) gene containing 6 coding exons encodes an ADP-ribosylhydrolase enzyme [8]. This enzyme eliminates the proteins’ post-translational addition of poly-ADP ribose (PAR) in cellular stress. Loss-of-function mutations in the *ADPRHL2* gene result in a recently defined disorder called stress-induced childhood-onset neurodegeneration with variable ataxia and seizures (CONDSIAS; OMIM: 618170) [9].

The CONDSIAS is an autosomal recessive disorder which its pertinent gene (*ADPRHL2*) is mapped on chromosome 1p35.3-p34.1. One frameshift, two nonsense, and three missense mutations have been reported in the association of this gene [10]. The phenotypes of this disorder have been reported as neurodegeneration, variable ataxia and seizures, tremor, nystagmus, balance problems, cerebellar, spinal cord and cerebral atrophy, hearing impairment and occasionally hearing loss, ptosis, ophthalmoplegia, dysarthria, muscle weakness, axonal neuropathy, dysmetria, and tongue fasciculation [10]. Symptoms and severity of the disorder appear to be different in patients and sometimes lead to early childhood death. In other words, although older patients present most of the above-mentioned symptoms, younger patients experience loss of developmental milestones and death in their early infancy [10].

In this study, we introduced a novel homozygous variant, c.636_639del: p.(Leu212fs), in a consanguineous Iranian family, which is associated with CONDSIAS disorder. Besides, novel imaging and clinical features are reported in this study. Future investigations, e.g. doing functional analysis, are necessary to validate the kinds of conclusions that can be drawn from this study.

## Case presentation

**Fig. 1:**
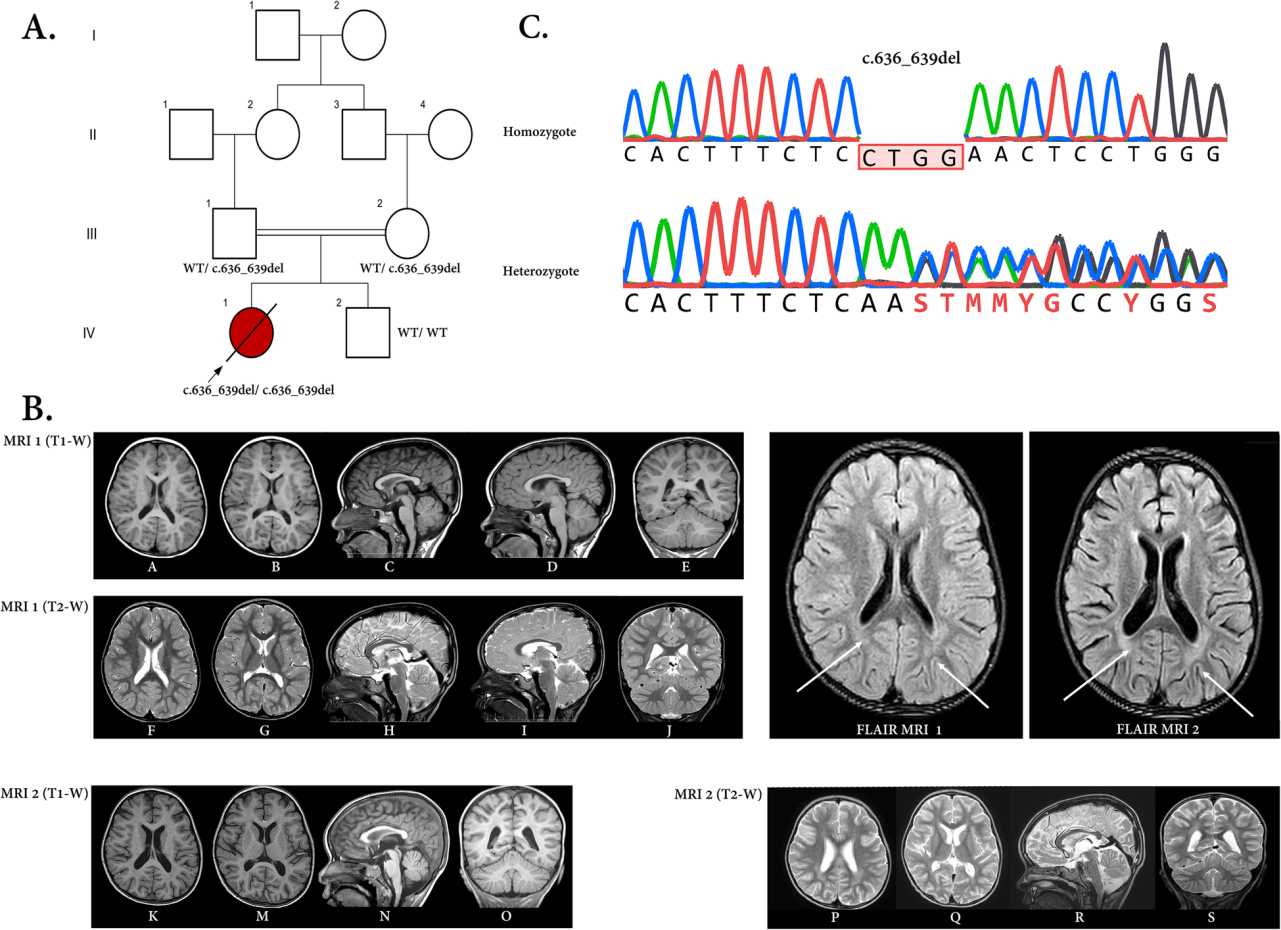
**a)** The pedigree of the family concerning the *ADPRHL2* variant and the pertinent chromatograms. The parents were heterozygote for the frameshift deletion in the *ADPRHL2* gene, while the patient (proband) was homozygote for this variant. The proband and her parents were subjected to whole-exome sequencing. In this pedigree, white symbols: unaffected who were homozygous for wild-type allele; red symbol: affected and homozygous for the variant; Square: male; circle: female; parallel lines: consanguineous marriage. Chromatograms showing nucleotide sequences of *ADPRHL2* in the cDNA regions of c.636_639. The gap region in ‘homozygote’ indicates the deletion from C to G (4 nucleotides) in the patient. **b)** Serial brain MRI without contrast of the patient with an interval of two months (MRI 1 and 2) shows progressive cerebral and cerebellar atrophy in T1-W (A-E, K-O) and their cognate T2-W images (F-J, P-S) in horizontal, sagittal, and coronal planes (compare A, B with K, M for cerebral and C, D with N, R for cerebellar atrophy). Besides, thin white arrows in FLIAR-MRI 1 and 2 exhibit progressive posterior deep white matter signal changes in two serial FLAIR sequences

The proband (IV.1) is a 4-year-old girl born to a consanguineous Iranian family (Fig. 1a) and was referred to the Children’s Medical Center hospital, Tehran, Iran, with different evident clinical symptoms including the new onset of general weakness, gait problem and variable ataxia, impaired speech, focal seizures, and progressive truncal hypotonia. All of the patient’s clinical information and the medical histories were collected at the Pediatric Neurology Division, Children’s Medical Center, Tehran University of Medical Sciences, Tehran, Iran.

The proband was born to an uneventful cesarean section with the birth-time weight of 3600 g. The birth-time measured head circumference was reported as normal. She was the first child of the family, the second child (IV.2), on the other hand, was completely normal. Family history was also negative for any diseases with similar phenotypes.

Following the tonsillectomy surgery, the proband gradually developed head nodding, upper limbs abnormal movements especially chorea, and then truncal hypotonia. She was also suffering from abdominal distention and food intolerance. Down the line of the surgery, she was referred to the hospital due to neurological deterioration. The early physical and neurological examination of the patient revealed a normal level of consciousness, normal eye contact but extraocular eye movements without any meaningful words production, truncal hypotonia with normal deep tendon reflexes, and weak gag reflexes. Feeding was conducted through nasogastric intubation (NG tube) at initial days of admission but gradually gastrointestinal (GI) intolerance of the patient became detectable and it was also found that she was suffering from severe abdominal distension. The seizure was controlled by administrating proper anti-seizure medications. Basic metabolic tests including serum ammonia, lactate, thyroid and liver function tests, blood gas analysis, serum amino acid chromatography, tandem mass spectrometry (MS/MS), urine organic acids profile were applied to the patient, however, all results were within normal limits. Because of suspected hearing problems, an auditory brainstem response (ABR) test was performed which revealed a severe bilateral sensorineural hearing loss in the patient, while the same condition had not been mentioned in each parent at all.

As early as the first week after the patient’s admission, the first brain magnetic resonance imaging (MRI) showed mild supratentorial and cerebellar atrophy with fine deep white matter signal changes in the posterior area (Fig. 1b; A-J and FLAIR-MRI 1-thin white arrows). Routine cerebrospinal fluid analysis (CSF) and CSF viral PCR for herpes simplex virus (HSV), Varicella zoster virus (VZV), enteroviruses, mumps, rubella, and measles viruses had not reported any abnormality. During the hospitalization, the patient was transferred to the pediatric ICU (PICU) section due to a gradually decreased level of consciousness and also cardiorespiratory problems nothing to say GI intolerance. She was admitted to PICU (more than two months) and intubated owing to a more decreased level of consciousness to the Glasgow Coma Scale (GCS) score 3. Anti-seizure medications were stopped due to concern about brain death according to EEG monitoring findings. Feeding was stopped through NG-tube due to progressive abdominal distension and total parenteral nutrition (TPN) was conducted to prevent nutritional imbalance. Second brain MRI that was done during PICU admission showed a progressive cerebral and cerebellar atrophy with more prominent deep white matter signal changes in posterior periventricular area (Fig. 1b; K-S and FLAIR-MRI 2-thin white arrows). To achieve an accurate molecular diagnosis, trio-whole-exome sequencing (WES) test was performed. Finally, the patient died due to cardiorespiratory arrest.

### Whole exome and sanger sequencing

For WES, a parent-offspring trio approach was used as previously described [11] with an average coverage depth of ~100X on Illumina Hiseq 4000 (Supplementary Method 1). To begin with, since the pattern of the pedigree was compatible with an autosomal recessive mode of inheritance, only variants that were heterozygous in unaffected parents and homozygous in the proband were considered. The WES data analysis was performed according to the previously reported works [12]. To be on the safe side, the analysis was applied according to the autosomal dominant mode of inheritance too.

Sanger sequencing in forward and reverse directions was performed to validate the candidate variant from WES and then co-segregation analysis was applied to the family. The primers were designed by Primer3.0 web-based server [13] to amplify the exon 4 of the *ADPRHL2* gene as follows: Forward: 5́-TCTGTCTCCCCTTCTGTTCC-3́ and Reverse: 5́-AGGGTCTGCAATTGAGGAAG-3́. The polymerase chain reaction (PCR) was performed in a standard condition [14]. The PCR products were sequenced by ABI 3500xl DNA Analyzer (Applied Biosystems Co., Foster City, CA), using the conventional capillary system; sequences were analyzed by the GenomeCompiler online tool (Twist Bioscience, USA) and Mutation Surveyor (v.3.24, SoftGenetics) [15] to identify any alternations (Fig. 1c).

**Fig. 2:**
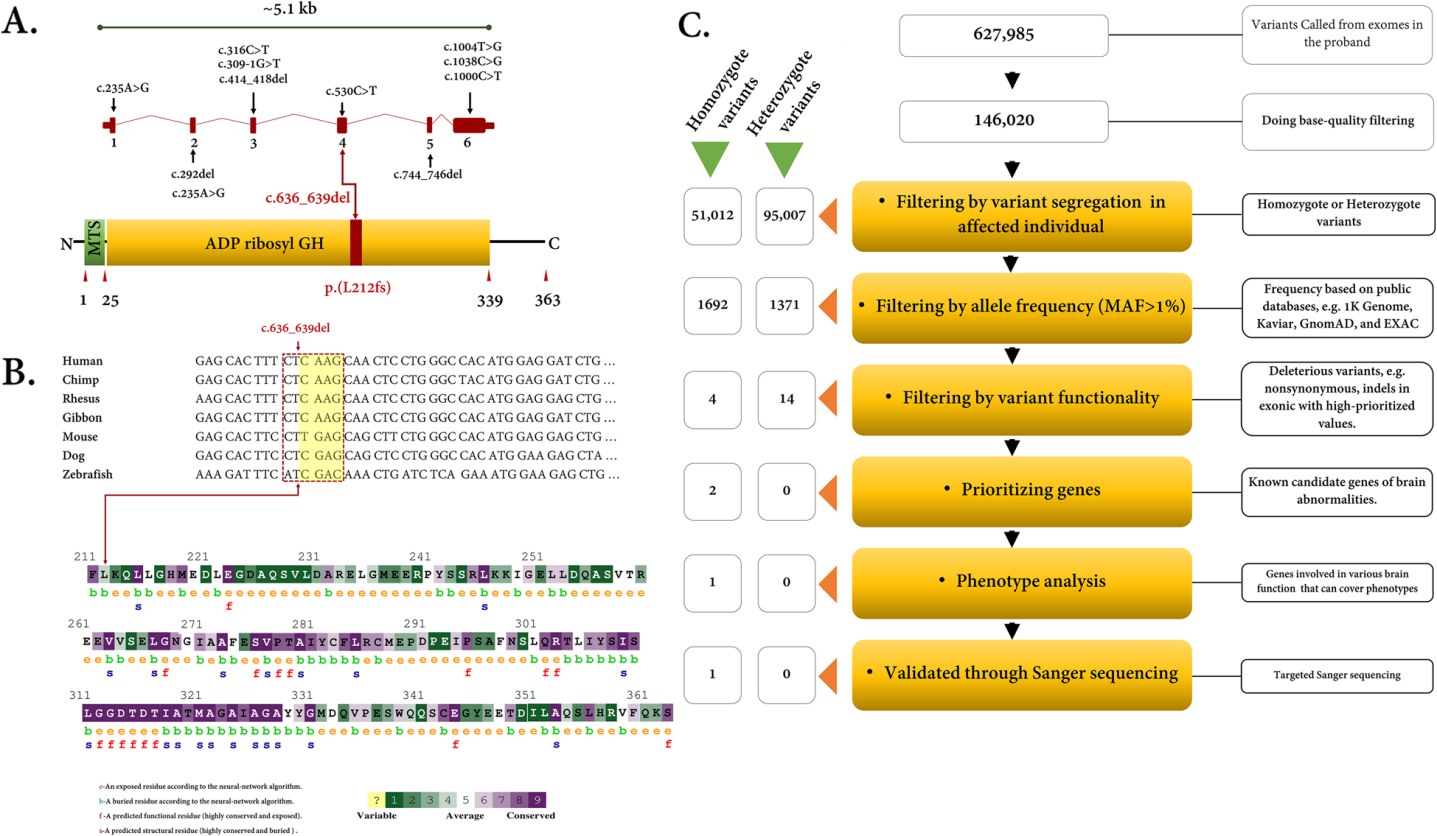
**a)** Structure of *ADPRHL2* gene (cDNA GenBank: NM_017825.2) with identified variants in exonic regions. The novel identified variant as the uncertain significance (VUS) (c.636_639del) in the *ADPRHL2* gene is illustrated in exon 4. The encoded protein involves 363 amino acids in two distinct domains: mitochondrial targeting sequence (MTS) and ADP ribosyl GH. The red section shows the location of the variant in the protein level. This variant causes loss of AA from the red section to the end of the C-terminal region. Other important identified variants are depicted in this figure. **b)** Nucleotide alignment shows high conservation of the codon residue in vertebrates which encodes protein AA resided after Leucine 212. The Yellow highlighted region indicates the deleted nucleotides. The ConSurf server was used to calculate conservation scores for the amino acid residues affected by the deletion. Scores ranged from 1 to 9, where an average score of 9 represents a highly conserved residue. ConSurf demonstrates evolutionary conservation profiles according to the phylogenetic relations between homologous sequences as well as amino acid’s structural and functional importance. **c)** Overview of variant filtering of whole-exome sequencing adapted to narrow down to most promising causative variants in the family. To show this that the possible variant might be inherited as dominant, the homozygote variants were filtered against but it did not result in any relevant variant which can justify the brain abnormality

Various databases were utilized to predict the overall score of pathogenicity of the variant, e.g. MutationTaster [16], ENTPRISE-X [17], Polyphen-2 [18], and Sorting Intolerant From Tolerant (SIFT) [19] (Supplementary Method 1). All tools used and also the achieved results are summarized in Table 1. The ConSurf [20] and UCSC databases were also used to provide an evolutionary conservation profile for ADPRHL2 in both protein and gene sequence levels to better judge the potential disrupting role of the variant (Fig. 2a and b). The frequency of the variant was checked in the same ethnic group using the Iranome (http://www.iranome.ir/) [21] as the local database.

**Table 1:** Several online databases used to predict the pathogenicity of p.(Leu212fs) in the family

Gene	NM	Exon	Alternation	dbSNP	MutationTaster	ENTPRISE-X	Provean	Polyphen	EXAC	1 k Genome	Iranome
*ADPRHL2*	NM_017825.2	4	c.636_639del; p.(Leu212fs)	NR	D	D	NA	D	N.R	N.R	N.R

*N****.****R* Not-Reported, *NA* Not Applicable, *D* Damaging

### Molecular findings

To show the underlying genetic cause(s), genomic DNA samples were analyzed by trio-WES and as a result, c.636_639del; p.(Leu212fs) homozygous frameshift deletion was confirmed by Sanger sequencing in the fourth exon of the *ADPRHL2* gene (Fig. 2a). The identified missense variant was not encountered in dbSNP version 147 [22], 1000 genome project phase 3 [23], Exome Sequencing Project (ESP) [24], ExAC [25], Iranome, Kaviar [26], Human Gene Mutation Database (HGMD) [27], and ClinVar database [28]. This variant was not found in the literature as well.

Finally, we reclassified the variant based on the American College of Medical Genetics and Genomics-Association for Molecular Pathology (ACMG-AMP guidelines [29]) into the variant of uncertain significance. Besides, by considering autosomal dominant inheritance, no candidate variant was resulted (Fig. 2c). The novel variant has been deposited to Leiden Open Variation Database (LOVD; https://databases.lovd.nl/shared/individuals/00299639) [30] and also ClinVar database (Accession Number: SCV001244198).

Several databases such as SIFT, Polyphen-2, and MutationTaster were used to evaluate the possible pathogenicity of the variant. All detailed results are described in Table 1. Furthermore, the conservation study by the ConSurf server showed an average conservation score (Average Score: ~ 5.1) for the involved residues (212–369). UCSC genome browser [31] was applied to check the conservation of the region at the nucleotide level. The result showed that the region is highly conserved, especially in primates. Amino acid and nucleotide alignments of ARH3 among higher vertebrates are depicted in Fig. 2b.

## Discussion and conclusions

**Fig. 3:**
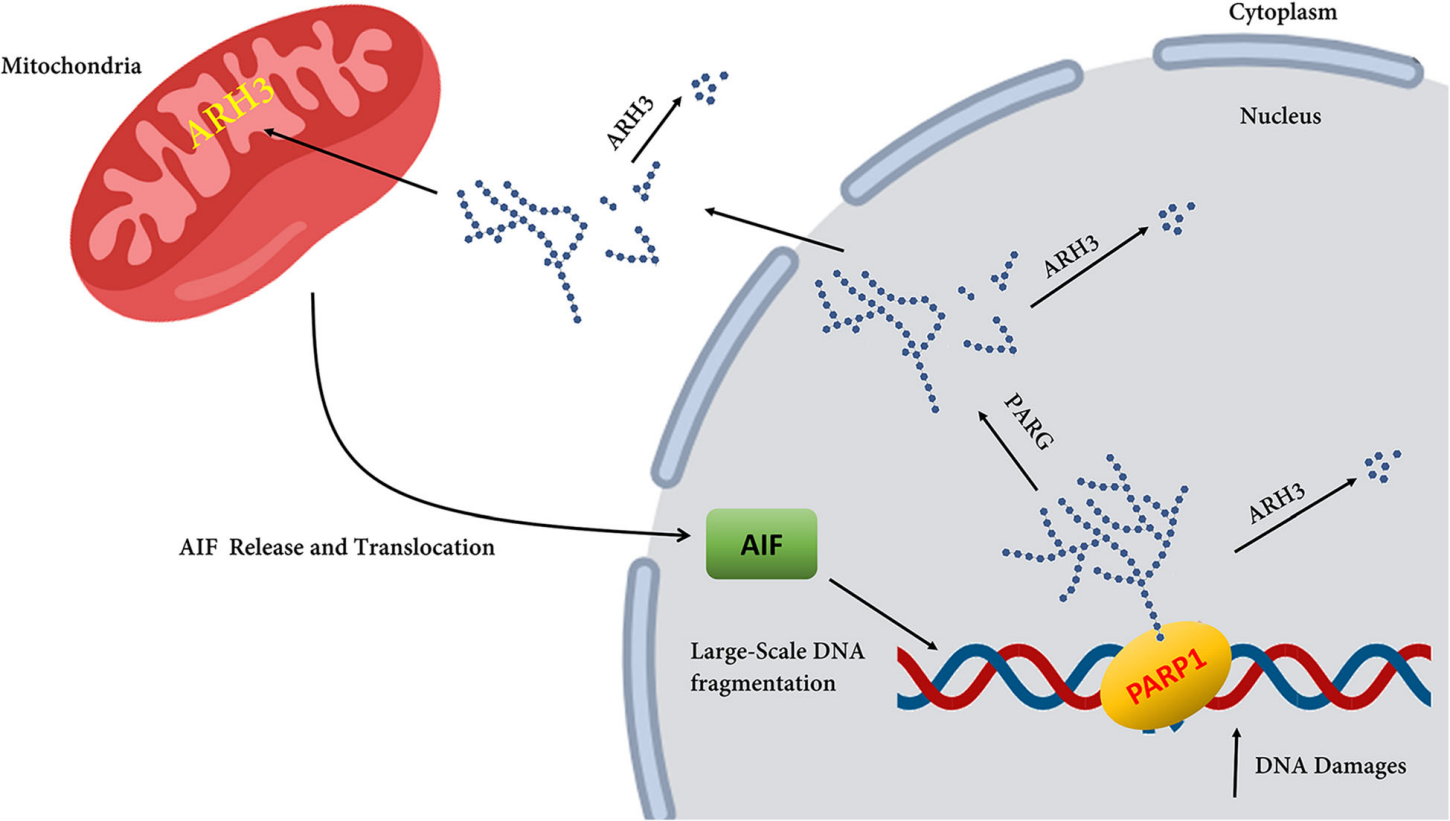
Model for the role of ARH3 (encoded by the *ADPRHL2* gene) in PAR degradation and apoptosis-inducing factor-mediated cell death. The PARP1 activation triggered by DNA damage leads to poly-ADP ribosylation of PARP1 and other acceptor proteins in the nucleus. Poly (ADP-ribose) glycohydrolase (PARG) hydrolyzes PAR added to the target protein, e.g. PARP1, hence facilitating the protein’s translocation to the cytoplasm and mitochondria. ARH3 hydrolyzes PAR. In the nucleus, AIF recruits various nucleases, e.g. cyclophilin A and H2AX, leading to large-scale DNA fragmentation. There is a general belief that ARH3 is also located in the mitochondrial matrix. The figure is redrawn from [32]

*ADPRHL2* (MIM: 610624; Gene ID: 54936) contains six coding exons that yield to a single protein-coding transcript, ADP-ribosylhydrolase 3 (ARH3) (Fig. 2a). The encoded protein is predicted to have a mitochondrial localization sequence (MTS) and a single enzymatic ADP-ribosyl-glycohydrolase (GH) domain (Fig. 3). This enzyme has been suggested playing an essential role in the cellular stress response pathway, e.g. DNA repair, transcription, telomere function, and apoptosis [32], and adding a poly-ADP-ribose (PAR) [33] to the target proteins. Hence, ADP-ribosylation can regulate protein function in a reversible post-translational modification. In humans, several ADP-ribose transferases, among them, ARH3 looms largely, transfer ADP-ribose from NAD^+^ to target proteins [34].

Mueller-Dieckmann et al. reported the three-dimensional structure of human ADP-ribosylhydrolase 3 (hARH3) and also suggested that its reversible activity is owing to the archetype of an all-alpha-helical protein fold [35]. The PARylated proteins can subsequently initiate cellular stress response pathways and after the resolution of the original insult, ADP-ribose polymers are rapidly removed (Fig. 3). Nevertheless, PAR modification can protect the cell from death [32], excessive PAR accumulation and/or failure to reverse PAR modification can pose the cell in danger of cell-death [36]. Humans have two genes encoding ubiquitously expressed PAR-degrading enzymes: *ADPRHL2* and *PARG* (MIM: 603501). Both can hydrolyze glycosidic bonds between ADP-ribose moieties and prevent PAR accumulation. It seems that inactivating mutations in both genes can increase the axonal cell-death [33], probably leading to white matter changes observed in the patient. Additionally, it has been identified that mitochondrial dysfunction can cause white matter damage/loss in patients [37], but the molecular mechanisms are still unclear.

As discussed, CONDSIAS is an autosomal recessive disorder caused by disruptive mutations in the *ADPRHL2* gene. For instance, six independent families carrying *ADPRHL2* mutations were reported [10] in them the exome sequencing identified different homozygous mutations including c.530C > T (exon 4), 5-bp deletion (exon 3), c.1000C > T (exon 6), c.235A > C (exon 2), c.100G > A (exon 1), and c.316C > T (exon 3) in the *ADPRHL2* gene (Table 2). All patients showed CONDSIAS common phenotypes. In 2018, Danhauser et al. also detected CONDSIAS disorder in 12 patients. The main reported clinical symptoms in those patients were a developmental delay, intellectual impairment, gait abnormalities, seizures, ataxia, neuropathy, sensorineural hearing loss, microcephaly, and respiratory insufficiency [9] (Table 2). Our case showed the related symptoms are in the line of Danhauser et al. report.

**Table 2:** The summary of reported cases of *ADPRHL2*-related neurodegenerative disorder and also a comparison with the present study

Study	Nationality of family	Number of patients	Sex (F/M)	Phenotype	Exon	Mutation	MRI findings
Ghosh et al.	United Arab Emirates	9	4F, 5 M	Childhood-onset, delayed motor development, impaired speech, intellectual disability (ID), stress-induced neurodegeneration, ataxia and seizures, progressive weakness, hypotonia with repeated pneumonia and cardiac arrest, ventilator-dependent at time of death, severe kyphoscoliosis, normal hearing but then developed severe sensorineural hearing loss (SNHL), profound type II muscle fiber atrophy	Exon 6	c.1000C > T	Mild cerebellar atrophy
	Italy	1	1 M	Childhood-onset, slow speech, normal motor development but then deteriorated by 2 years, stress-induced neurodegeneration, ataxia, and seizures, normal intellect but then started deteriorating at age 11 years, myopathic changes on muscle biopsy (at age of 11 years)	Exon 3	c.316C > T	Cerebellar vermis atrophy
	Turkey	1	1F	Childhood-onset, normal speech, motor and intellectual development, stress-induced neurodegeneration, ataxia, and seizures, claw hand and pes cavus deformities, scoliosis, SNHL at 10 years, tracheotomy, ventilator support	Exon 2	c.235A > C	Mild cerebellar atrophy, spinal cord atrophy
	Pakistan	2	2F	Childhood-onset, normal speech and motor development but then deteriorated by 2 years, mild ID, stress-induced neurodegeneration, ataxia, and seizures, asthma	Exon 3	5-bp (c.414- 418delTGCCC)	Mild cerebellar atrophy
	Iran	2	1F, 1 M	Childhood-onset, speech only a few words, normal motor development but then deteriorated, normal intellect but then stagnated, stress-induced neurodegeneration, ataxia and seizures, progressive weakness, tremors, frequent falling, progressive external ophthalmoplegia	Exon 4	c.530C > T	Female: normal (3 years) Male: N/A
	Turkey	1	1F	Childhood-onset, normal motor but delayed speech development, mild ID, stress-induced neurodegeneration, ataxia and seizures, distal muscle atrophy, pes cavus deformity, toe abnormality, scoliosis, brisk deep tendon reflexes (DTRs), positive Babinski reflex, intentional tremor	Exon 1	c.100G > A	Mild cerebellar vermis atrophy, spinal cord atrophy
	Germany	2	1F, 1 M	Childhood-onset, developmental delay, and ID, stress-induced neurodegeneration, gait abnormalities, ataxia and seizures, facial myoclonia, diplopia, neuropathy, respiratory insufficiency	Exon 6	c.1004 T > G	Male: N/A, Female: Basal ganglia, cortex and cerebellum involvement
	Lebanon	1	1F	Childhood-onset, developmental delay, and ID, stress-induced neurodegeneration, gait abnormalities, ataxia and seizures, neuropathy, facial myoclonia, nystagmus, respiratory insufficiency	Exon 5	c.744_746del	Corpus callosum, basal ganglia, cortex and cerebellum involvement
	N/A	1	1F	Childhood-onset, developmental delay, and ID, stress-induced neurodegeneration, gait abnormalities, ataxia and seizures, neuropathy, SNHL, strabismus, microcephaly	Exon 6	c.1038C > G	Corpus callosum and cerebellum involvement
Danhauser et al.	N/A	2	1F, 1 M	Childhood-onset, stress-induced neurodegeneration with variable ataxia, gait abnormalities, nystagmus, strabismus, respiratory insufficiency	Exon 6	c.1004 T > G	Cerebellum involvement
	Kosovo	1	1 M	Childhood-onset, developmental and intellectual impairment, stress-induced neurodegeneration, ataxia, gait abnormalities, putative external ophthalmoplegia with ptosis, impaired saccades, and upward gaze and nystagmus, putative retinal pigment epithelium anomalies, neuropathy, microcephaly	Exon 6	c.1004 T > G	Cerebellum involvement
	Poland	1	1F	Childhood-onset, developmental delay and ID, stress-induced neurodegeneration, ataxia and seizures, gait abnormalities, neuropathy	Exon 6	c.1004 T > G	N/A
	China	2	2F	Childhood-onset, developmental delay, and ID, stress-induced neurodegeneration, ataxia and seizures, gait abnormalities, neuropathy, respiratory insufficiency	N/A	c.309-1G > T	Cerebellum involvement
	Turkey	2	1F, 1 M	Childhood-onset, developmental delay, and ID, stress-induced neurodegeneration, ataxia and seizures, gait abnormalities, microcephaly, respiratory insufficiency	Exon 2	c.292delG	Male: Basal ganglia involvement
The Present study	Iran	1	1F	Childhood-onset, normal development at first, stress-induced neurodegeneration, frequent falling down, imbalance gait and ataxia, general motor weakness and truncal hypotonia, focal seizures, impaired speech, and progressive, severe abdominal distension and GI intolerance, cardiorespiratory problems, SNHL	Exon 4	c.636_639del	Mild supratentorial atrophy, progressive cerebral and cerebellar atrophy

**Fig. 4:**
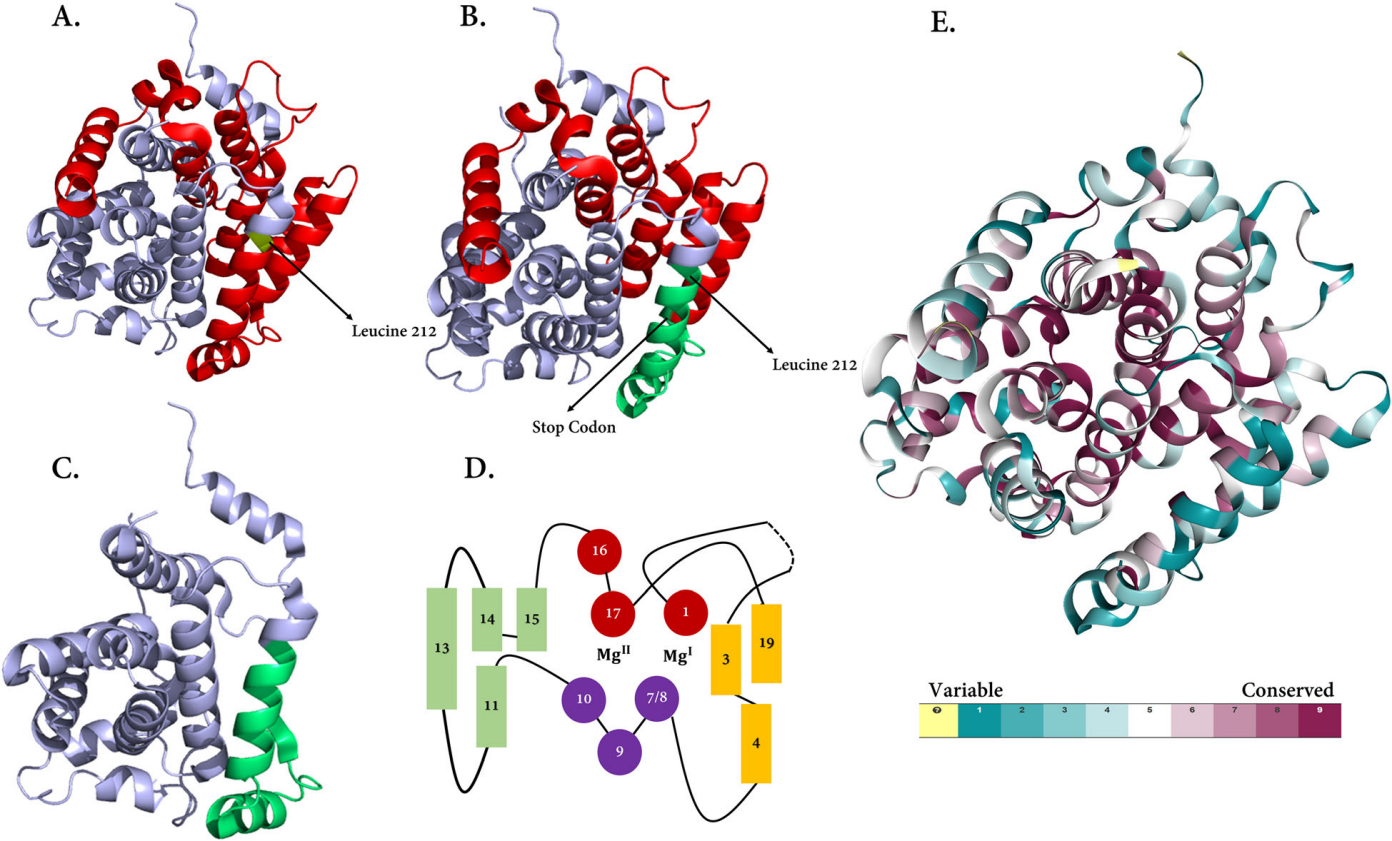
ARH3 predicted structure. **a)** structure modeling of the normal protein and superimposed structure modeling of the variated protein was based on a well-known template (PDB: 2FOZ); the variated site of p.Leu212 is highlighted in greenish-yellow color. The red sections are the affected parts that will be deleted because of the premature stop codon. A BLAST sequence search against the protein data bank (PDB) was performed to select the template structure with the most sequence similarity to the domain of ARH3. To further evaluate, we used “human ADP-ribosylhydrolase 3” as the favorite template (PDB ID: 2FOZ) [35]. To predict any impact of p.(Leu212fs) on the structure and function of ARH3, the Iterative Threading Assembly Refinement (I-TASSER) package (https://zhanglab.ccmb.med.umich.edu/I-TASSER/) was utilized in which the highest significant alignment regions of the templates were selected by considering the Z-score measurement. The protein structure and possible effects of the novel variant on protein structure were depicted by the PyMOL package (https://pymol.org/). **b)** the premature stop codon is shown, while the remained parts are exhibited in green. **c)** the remained parts after the deletion of amino acids are shown. **d)** two Mg^2+^ ions are indicated. α-Helices approximately perpendicular to the viewing plane are represented as circles, those oriented roughly horizontally in the viewing plane as rectangles. Because of the frameshift deletion, helixes from 13 to 19 will be deleted. This figure is redrawn from [35]. **e)** The amino acid sequence of ARH3 colored based on conservation scores derived from the ConSurf database; highly conserved regions are shown in purple, although weak conserved areas are indicated in blue. The highly conserve areas make the binding site and also an active domain of ARH3

In our case, we found a homozygous frameshift variant in the *ADPRHL2* gene (c.636_639del: p.(Leu212fs)) which has not been reported in pieces of literature. We localized this variant to an α-helical loop between 11th and 13th α-helixes within the ADP-ribosylhydrolase domain which takes a center stage in forming the substrate-binding site, defined by the position of two Mg^2+^ ions located in adjacent binding sites; therefore, the variant is predicted to affect the protein structure and its enzymatic activity as well (Fig. 4 a-e). In other words, the variation identified in this study causes a premature termination codon (246th codon) in the 4th exon of *ADPRHL2*. It, therefore, seems probable that an altered protein will be expressed but with the truncated C-terminal region which has a detrimental effect on enzyme activity. The phenotype observed may, thus, be a result of the loss of function due to the altered protein inhibiting the enzyme activity of ARH3. This variant was considered a strong candidate for the pathogenic variant in this family.

Medical assessment of the patient, 4-year-old girl, revealed various symptoms including variable ataxia and seizures, impaired speech, and bilateral sensorineural hearing loss. These findings were consistent with previously reported cases in that the most common features were encompassing normal or delayed development, intellectual disability, progressive motor and speech regression, muscle weakness, ataxia and gait problem tongue fasciculation, seizures, sensorineural hearing loss, and neuropathy. Some other extra-neurologic symptoms are respiratory insufficiency, asthma, and ophthalmologic problems.

Severe gastrointestinal intolerance (GI) and abdominal distension was a novel extra-neurologic finding in IV.1 so that she could not be able to tolerate even minimal amounts of liquid diet during admission at PICU, therefore nutrition of the patient was continued through total parenteral nutrition (TPN). According to previous studies, it sounds that hypoactivation of ADPRHL2 has similar effects on the mitochondria as PARP1 hyperactivation. GI intolerance has been reported as a common manifestation of mitochondrial-based disorders [38]. Furthermore, PARP1 hyperactivation (accumulated PAR) has been reported to tightly associated with inflammation in the gastrointestinal system. *PARP-1*^*−/−*^ animals displayed a significantly lower level of intestinal inflammation [39]. In this study, we hypothesized that the c.636_639del mutation could be categorized as the loss-of-function mutation, which potentially leads to PAR accumulation. Thus, gastrointestinal manifestation could be probable. Clinically, to find the probable cause of GI intolerance in the patient, we carried out the upper GI endoscopy, and also performed common gastroenterological tests including stool PH test, faecal (fecal) calprotectin, and routine microbiology tests. None of them showed any abnormalities.

The most common neuroimaging findings that have been stated in association with CONDIAS disorder are cerebral and cerebellar atrophy, progressive cerebellar vermis atrophy, basal ganglia, corpus callosum, and spinal cord involvement but cerebral white matter signal changes have not been reported. Therefore, another phenotypical novelty of the patient was progressive deep white matter signal changes in the occipital area of the brain which has not been reported in neuroimaging findings of CONDIAS disorder. To impute the white matter changes to the mutation in ADPRHL2, we excluded other possible causes, e.g. circulatory changes, any possible systemic hypotension during and/or after hypoxia (asphyxia), a positive history of infection (inflammation), and mechanical problems during delivery resulting in excessive molding and depression of the skull. Thus, to our knowledge, this study extends both clinical and neuroimaging findings of this disorder.

The detection of an *ADPRHL2* variation in this family asserts that the ARH3 plays an important role in the brain. This study also demonstrates the effectiveness of WES technology for detecting causative gene defects. In this study, a frameshift deletion at the important part of ARH3 protein, ADP ribosyl GH domain, was introduced as a cause of CONDIAS. The phenotypes observed were consistent with the reported cases, however, we detected some novel phenotypes as well, e.g. progressive deep white matter signal changes in the occipital area and cerebral white matter of the brain, gastrointestinal intolerance, and abdominal distension. Future investigations, i.e. doing functional analysis, are necessary to validate some kind of conclusions drawn from this study.

In a nutshell, the present study underscores the usefulness of WES in finding the genetic basis of neurological disease. The upshot of this is the possibility that the c.636_639del variant in exon 4 of *ADPRHL2* can make encoded protein to malfunction. Nevertheless, before using this data in genetic counseling, we strongly suggest doing functional analysis.

## Supplementary information

**Additional file 1.**

## Data Availability

The datasets analysed during the current study are available from the corresponding author on reasonable request. The novel variant has been submitted to the specific gene variant database Leiden Open Variation Database (LOVD; individual number: 00299639; https://databases.lovd.nl/shared/individuals/00299639) and ClinVar database (accession number: SCV001244198; https://www.ncbi.nlm.nih.gov/clinvar/53619267).

## Abbreviations

CONDSIAS: Stress-induced childhood-onset neurodegeneration with variable ataxia and seizures
ADPRHL2: ADP-Ribosylhydrolase Like 2
ARH3: ADP-ribosylhydrolase enzyme
PAR: Poly-ADP ribose
GI: Gastrointestinal intolerance
WES: Whole-exome sequencing
NG tube: Nasogastric intubation
PICU: Pediatric ICU
GCS: Glasgow Coma Scale
ESP: Exome Sequencing Project
PDB: Protein data bank
I-TASSER: Iterative Threading Assembly Refinement
