# Supplementary material for: Novel imaging and clinical phenotypes of CONDSIAS disorder caused by a homozygous frameshift variant of ADPRHL2: a case report
Source: BMC Neurol. 2020 Aug 3;20:291. doi: 10.1186/s12883-020-01873-3 (PMC7397971; doi:10.1186/s12883-020-01873-3)
Supplement: Supplementary file 1 — Additional file 1. [file 12883_2020_1873_MOESM1_ESM.doc]

**Supplementary Methods**

**Full methods of whole exome sequencing (WES)**

***1. Construction of pre-enrichment DNA libraries***

Pre-enrichment DNA libraries were constructed using the Illumina TrueSeq DNA sample preparation kit (Illumina, Inc., San Diego, CA, USA). For this purpose, the genomic DNA was isolated, and the qualified samples were randomly fragmented into the fragments were between 200 and 300 bp. Thence, adapters were ligated to both ends of the fragments and the adapter-ligated templates were purified using AMPure XP beads (Beckman **Coulter, Inc.,** Brea, CA, USA).

***2. Exome enrichment***

Approximately a total of 0.5 µg of each library was pooled. DNA was hybridized overnight using Illumina TrueSeq Exome Enrichment probes (Illumina, Inc.). Then, the biotinylated probes and the hybridized sample DNA were captured via streptavidin beads and rinsed in a three-step process, and then DNA was eluted from the probes. The above processes were repeated one more time on eluted DNA. Captured DNA fragments containing ligated adapter sequences were enhanced via PCR using adapter-specific primers. The DNA was then isolated using AMPure XP beads and analyzed using the Agilent Bioanalyzer 2100 (Agilent Technologies, Inc., Santa Clara, CA, USA).

***3. Cluster generation of the enriched exome libraries***

The enriched exome libraries were loaded onto flow cells for cluster generation using a TrueSeq PE Cluster Kit v3-cBot-HS (Illumina, Inc.). One lane of each flow cell was reserved for a PhiX control.

***4. Exome sequencing***

The flow cells with the clusters of the enriched exome libraries were then transferred to HiSeq4000 (Illumina, Inc.). High-throughput sequencing was performed for each captured library to ensure that each sample met the desired average sequencing depth of at least 40X.

***5. Alignment of sequences***

Raw reads in FASTQ format from the exome sequencing were aligned to the hg19 reference genome downloaded from UCSC with BWA (bwa-0.5.9; BWA is a software package for mapping low-divergent sequences) using a seed length of 45 as the default parameter. The aligned reads were processed, and PCR duplicates were removed with the Sequence Alignment/Map (SAM) format (SAMtools-0.1.16) (http://samtools.sourceforge.net) and processed with the Count Covariates, Table Recalibration, Realigner Target Creator, Indel Realigner step with GATK (GenomeAnalysisTK-1.4) (Broad Institute, Inc. Cambridge, MA, USA).

***Detection of single nucleotide variants (SNVs) and insertions or deletions (Indels)***

SNVs and Indels were detected with GATK according to the previous reports [1].

***Annotation of SNVs and Indels***

(1) **Annotation by genomic regions**: The SNVs and Indels were annotated by genomic regions, e.g. intergenic, 5′-UTR, intron, coding sequence (CDS), splicing, and 3′-UTR. We analyzed the SNPs and Indels of CDS, the promoters, the splice sites, the 3′-UTR, the 5′-UTR, and the splice sites close to the intron (±20) region.

(2) **Annotation by functional effects**: We analyzed the effect of the variants on the codons with SNVs in the CDS annotated as synonymous, missense, nonsense, and read-through with In-house script. The Indels in the CDS were also annotated by whether they caused frame-shift mutations or not. We used *in-silico* prediction using Sorting Tolerant from Intolerant (SIFT; Score<0.05), MutationTaster (Score>0.5), and Combined Annotation Dependent Depletion (CADD; score >30) to predict deleterious effects and PolyPhen 2 to predict probably or possibly damaging effects.

(3) **Annotation by inheritance models**: As we performed the solo-WES for the patient, dominant (de novo) and recessive inheritance models were hypothesized. For each inheritance model, candidate variants were identified through an optimal prioritization process as follows: Known candidate variants for brain abnormality were extracted by comparison with data from the *Online Mendelian Inheritance in Man (OMIM)* database Common variants among the remaining SNVs and Indels were excluded by comparison with data from the 1000 genomes reported in dbSNP v147 and from an in-house database (Iranome [2]). All statistics are summarized in figure 2c.

**References**

1. Spencer, D.H., et al., *Performance of Common Analysis Methods for Detecting Low-Frequency Single Nucleotide Variants in Targeted Next-Generation Sequence Data.* The Journal of Molecular Diagnostics, 2014. **16**(1): p. 75-88.

2. Fattahi, Z., et al., *Iranome: A catalog of genomic variations in the Iranian population.* Human mutation, 2019. **40**(11): p. 1968-1984.
